# Insight into the formation of trumpet and needle-type leaf in *Ginkgo biloba* L. mutant

**DOI:** 10.3389/fpls.2022.1081280

**Published:** 2022-12-09

**Authors:** Fang Tang, Pengbo Sun, Qian Zhang, Fengwei Zhong, Ying Wang, Mengzhu Lu

**Affiliations:** ^1^ State Key Laboratory of Tree Genetics and Breeding, Key Laboratory of Tree Breeding and Cultivation of The National Forestry and Grassland Administration, Research Institute of Forestry, Chinese Academy of Forestry, Beijing, China; ^2^ Co-Innovation Center for Sustainable Forestry in Southern China, Nanjing Forestry University, Nanjing, China; ^3^ Taishan Academy of Forestry Sciences, Tai’an, China; ^4^ State Key Laboratory of Subtropical Silviculture, School of Forestry and Biotechnology, Zhejiang A&F University, Hangzhou, China

**Keywords:** *Ginkgo biloba* L., leaf shape regulation, anatomical structure, transcriptome analysis, adaxial-abaxial polarity, leaf boundary

## Abstract

The leaf type of a plant determines its photosynthetic efficiency and adaptation to the environment. The normal leaves of modern *Ginkgo biloba*, which is known as a “living fossil” in gymnosperm, evolved from needle-like to fan-shaped with obvious dichotomous venation. However, a newly discovered *Ginkgo* variety “SongZhen” have different leaf types on a tree, including needle-, trumpet-, strip-, and deeply split fan-shaped leaves. In order to explore the mechanism in forming these leaf types, the microscopy of different leaf types and transcriptome analysis of apical buds of branches with normal or abnormal leaves were performed. We found that the normal leaf was in an intact and unfolded fan shape, and the abnormal leaf was basically split into two parts from the petiole, and each exhibited different extent of variation. The needle-type leaves were the extreme, having no obvious palisade and spongy tissues, and the phloem cells were scattered and surrounded by xylem cells, while the trumpet-type leaves with normal vascular bundles curled inward to form a loop from the abaxial to adaxial side. The other type of leaves had the characteristics among needle-type, trumpet-type, or normal leaves. The transcriptome analysis and quantitative PCR showed that the genes related to abaxial domain were highly expressed, while the adaxial domain promoting genes were decreasingly expressed in abnormal-type leaf (ANL) buds and abnormal leaves, which might lead to the obvious abaxialized leaves of “SongZhen.” In addition, the low expression of genes related to leaf boundary development in ANL buds indicated that single- or double-needle (trumpet) leaves might also be due to the leaf tissue fusion. This study provides an insight into the mechanism of the development of the abnormal leaves in “SongZhen” and lays a foundation for investigating the molecular mechanism of the leaf development in gymnosperms.

## Introduction

The leaf is the main organ for plants to receive light energy and absorb carbon dioxide to produce carbohydrates. It is the most sensitive and plastic organ to environmental changes, and its morphological structure evolves and changes under different ecological conditions to adapt to the environment (Chitwood and Sinha, 2016). The earliest vascular plants had no leaves at all, only sparse stomata distributed on the stems. The flat megaphyll leaves with vein first appeared in the Devonian period 360 million years ago (Kenrick and Crane, 1997). The generation of flat leaves is an important time point in the evolution of leaves because flat and stretched leaves greatly improve the area to receive light, thus increasing the efficiency of carbon fixation, which plays an important role in the dominant position of giant leaf plants in many ecosystems (David, 2005). The development of leaf is mainly divided into three processes: leaf primordium initiation, leaf polarity establishment, and leaf expansion (Dkhar and Pareek, 2014). When leaf development initiates at the peripheral zone (PZ), which surrounds the central zone of the shoot apical meristem (SAM), a boundary region partitions the new leaf primordium from the SAM on the adaxial side. Subsequently, the leaf primordia undergo asymmetric division and differentiation along the adaxial–abaxial, medio–lateral, and proximal–distal axes to form leaf organs (Du et al., 2018). The abnormal development of adaxial–abaxial axis will cause leaf curl, leading to the reduced area of light acceptance and even plant death. Adaxial–abaxial polarity is maintained and further strengthened *via* adaxial- and abaxial-promoting genes, encoding transcription factors, and small RNAs that act in conserved and partially redundant pathways (Bar and Ori, 2014; Du et al., 2018).

The study on adaxial–abaxial axis of leaves began from the mutants of *PHANTASTICA* (*PHAN*) in *Antirrhinum*. The adaxial side of leaves of the mutants showed an abaxial character, and the severe mutants even had needle-like leaves, which revealed that *Phan* gene promotes the differentiation of adaxial tissues of leaves (Waites and Hudson, 1995). Although *PHAN* and its orthologues are uniformly expressed in young leaf primordia of respective species, their roles in adaxial specification are not strictly conserved. The knockdown of *PHAN* orthologues can lead to abaxialization in tomato (*LePHAN*) (Kim et al., 2003) but not in maize (*RS2*) (Timmermans et al., 1999) or *Arabidopsis* (*AS1*) (Byrne et al., 2000). In addition, *KANADI* (*KAN*) gene, encoding a transcription factor containing MYB-like GARP DNA binding domain, promotes abaxial domain (Kerstetter et al., 2001). *YABBY* (*YAB*) genes function relatively later in leaf development and are considered to act downstream of the *KAN*. In *Arabidopsis*, *YAB* genes are expressed in the abaxial domain and redundantly promote abaxial identity (Siegfried et al., 1999; Eshed et al., 2004). Antagonistically, the adaxial expression of homeodomain/leucine-zipper (HD-ZIP) genes, namely, *PHABULOSA* (*PHB*), *PHAVOLUTA* (*PHV*), and *REVOLUTA* (*REV*), are sufficient to define adaxial cell fate (Emery et al., 2003), but they are regulated by miR165 and miR166 (Tang et al., 2003). Gain-of-function *HD-ZIPIII* mutants and resistance to the inhibition by miR165/166 lead to the formation of adaxialized leaves, whereas loss-of-function *phb phv rev* triple-mutant plants form abaxialized leaves and exhibit loss-of-SAM phenotypes (Wenkel et al., 2007; Kim et al., 2008). The auxin signaling pathway is also involved in the regulation of adaxial–abaxial axis of leaves. Three redundant repressive AUXIN RESPONSE FACTORs (ARFs), namely, *ETTIN* (*ETT* or *ARF3*), *ARF4*, and *ARF2*, are expressed in the abaxial domain and promote abaxial cell fate (Pekker et al., 2005; Guan et al., 2017). The mutual antagonism exists between regulatory factors of the adaxial and abaxial domains of leaves. The adaxially localized ASYMMETRIC LEAF1 (AS1) and AS2 complex negatively regulates the expression of *ETT* and *ARF4* (Iwasaki et al., 2013) and also directly inhibits MIR166A and *YAB5* expression in the adaxial domain (Husbands et al., 2015). In the abaxial domain, KAN1 binds to the promoter regions of MIR166A and MIR166F and downregulates the expression of MIR166F but not MIR166A (Merelo et al., 2013), implying that complicated and fine-tuned regulations exist. In addition, ARF and KAN may form complexes and play a synergism role in promoting abaxial tissue differentiation (Kelley et al., 2012).

The formation of boundaries plays an important role in the development of plant lateral organs, and some transcriptional regulators are involved in the establishment and maintenance of boundaries (Žádníková and Simon, 2014). The CUP-SHAPED COTYLEDON (CUC) NAC-domain transcription factors *CUC1*, *CUC2*, and *CUC3* are specifically expressed in boundary positions, and mutations of *CUC* genes lead to varying degrees of shoot organ fusion phenotypes due to the failure of boundary formation (Aida et al., 1999; Vroemen et al., 2003). The class II TEOSINTE BRANCHED1/CYCLOIDEA/PROLIFERATING CELL FACTOR (TCP) transcription factors can regulate plant organ morphology by participating in cell proliferation and differentiation. They repress marginal meristem activity and control the morphogenesis of shoot lateral organs by negatively regulating the expression of boundary-specific genes. Gain of function of *TCP3* suppresses the expression of *CUC* genes and results in the fusion of cotyledons and defects in the formation of shoots (Koyama et al., 2007). The WUSCHEL-RELATED HOMEOBOX (WOX) transcription factors can regulate blade expansion specifically along the medio-lateral axis. PRESSED FLOWERS (PRS) and WOX1 redundantly promote lateral lamina outgrowth, and the *wox1 prs* double mutant produces narrow leaves with disturbed polarity but unaltered leaf length (Vandenbussche et al., 2009; Nakata et al., 2012). In addition, NGATHA (NGA) and TCP transcription factors redundantly inhibit *WOX* expression to terminate the marginal meristem in *Arabidopsis* (Alvarez et al., 2009).

*Ginkgo biloba* L. belongs to Ginkgoaceae and *Ginkgo* genus, one of the oldest living gymnosperms left over from the Quaternary glacier movement, thus also known as “living fossil.” Leaves in Ginkgoales tend to be laminated and petiolate due to planation, webbing, and fusion of telomes and mesomes (Zhou, 1991; Zhou, 2003). *Ginkgo* originated from the middle Carboniferous period of Paleozoic, which was dichophyllum with two main veins, and then evolved into *Trichopitys* having hairy leaves with four main veins in the Late Permian Period. The hairy leaves gradually became webbing, evolved into thin lobed type *Baiera*, and then into the four lobes type *Ginkgoites* sp. There were two branches of evolution that followed: one was *Ginkgoites pluripartita* in the cretaceous period of Mesozoic Era, which was totally extinct in Quaternary Period; the other developed into modern *G. biloba* having the fan-shaped leaves without cracks (Zhou and Zheng, 2003; Zhou, 2006). The ancient plants with megaphyll leaves had obvious dichotomous venation, but today, *G. biloba* still retains this ancient venation feature (Beerling et al., 2001). The leaf shape of the tertiary *Ginkgo* fossils unearthed in North Dakota is consistent with that of today’s *G. biloba* (Zhou et al., 2012). “SongZhen” (pine needle) is a recently discovered *G. biloba* variety with many different leaf types in a tree. Besides the normal fan-shaped leaves, needle-, trumpet-type, and deeply split leaves sprout on one or different branches of “SongZhen” (Wang et al., 2009).

In order to explore the mechanism in the formation these leaf types of “SongZhen,” microscopy observation of different leaf shapes and transcriptome analysis of apical buds of branches with normal and abnormal leaves were performed in this study. It was found that the obvious abaxialization could be the reason to form the “SongZhen” leaves. The expression of abaxial domain-promoting genes were higher, and the adaxial domain-promoting genes were lower in the abnormal-type leaf (ANL) buds, which might lead to the obvious abaxialized leaves of “SongZhen.” In addition, the expression of genes related to leaf boundary development was generally low in ANL buds, indicating that the abnormal-type leaves of “SongZhen” may be also due to the leaf tissue fusion. These results lay a foundation for studying on the molecular evolution and regulation of the development of leaves in gymnosperms.

## Materials and methods

### Plant materials

The plant material used in this study is a 4-year-old *G. biloba* “SongZhen,” which grew in a clonal plantation located in Tai’an, Shandong province, China. “SongZhen” is a new *Ginkgo* variety that was discovered and cultivated by Tai’an City Academy of Forestry Science (Wang et al., 2009). In the middle of April, when Ginkgo leaf buds were formed, about 120 apical buds of normal leaf (NL) and abnormal-type leaf (ANL) branches were collected separately with RNase-free blades and immediately frozen and stored in liquid nitrogen. The samples were divided into three parts as three biological repeats for transcriptome analysis. When the leaves of “SongZhen” were expanded, the different types of young leaves from ANL and NL branches were collected for quantitative PCR.

### Sample collection and paraffin section of leaf

After the leaves were fully expanded, the representative leaves of different leaf types of “SongZhen” were collected. Among them, the fusion and division region of abnormal-type leaves and the widest sector of the unfolded leaves were prepared for cross-sections using paraffin cutting (He et al., 2014). The section thickness was <10 µm and stained with Safranin O-Fast Green for light microscope observation. The stained sections were observed and photographed under a Olympus BX51 microscope, and cellSens standard software was used to splice a complete image for long or large samples.

### Total RNA extraction

The apical buds from NL and ANL branches were mixed in equal amount separately to extract the total RNAs using Total RNA Purification Kit (LC sciences, #TRK-1001) following the manufacturer’s instructions. Briefly, 100 mg of tissues was grinded into powder in liquid nitrogen; then, 600 µl of extraction buffer with 6% Plant RNA Isolation Aid (Ambion, #Am9690) was added. The mixture was shaken vigorously, then incubated on ice for 15 min and centrifuged at 12,000 rpm for 10 min at room temperate, by which the yield of total RNA could be improved.

### High-throughput sequencing and quality control

The cDNA libraries from the above total RNA samples and high-throughput sequencing were completed by BioMarker (BMK, Beijing). Sequencing libraries were generated using NEBNext UltraTM RNA Library Prep Kit for Illumina (NEB, USA) following the manufacturer’s recommendations, and index codes were added to attribute sequences to each sample. Briefly, mRNA was purified from total RNA using poly-T oligo-attached magnetic beads and then was randomly broken into short fragments by fragmentation buffer. AMPure XP bead was selected for fragment selection, and the final sequencing library was obtained by PCR amplification. After the library passed the quality inspection, it was sequenced with Illumina NovaSeq 6000, and the length of the sequenced reading was 2×150 bp (PE150). The RNA-Seq raw data had been submitted to the NCBI Sequence Read Archive under BioProject accession number PRJNA896381. The clean data were obtained by removing reads containing adapter, reads containing poly-N, and low-quality reads from raw data using fastp software. At the same time, Q20, Q30, GC content, and sequence duplication level of the clean data were calculated. All the downstream analyses were based on clean data with high quality.

### Genome comparison and expression analysis

These clean reads were aligned with *G. biloba* genome (Guan et al., 2016) by HISAT2 software, and the allowed number of mismatch nucleotide was set to “0” or “1.” StringTie was used for transcripts assembly and new transcripts prediction, and the alternative splicing type and corresponding expression amount of each sample were obtained through ASprofile software. Quantification of gene expression levels were estimated by fragments per kilobase of transcript per million fragments mapped (FPKM). Differential expression analysis of NL and ANL buds was performed using the DEseq. The FDR < 0.05 and fold change ≥2 was set as the threshold for significant differential expression. Gene Ontology (GO) enrichment analysis of the differentially expressed genes (DEGs) was implemented by the GOseq R packages based Wallenius non-central hyper-geometric distribution (Young et al., 2010), and KOBAS (Mao et al., 2005) software was used to test the statistical enrichment of DEGs in Kyoto Encyclopedia of Genes and Genomes (KEGG) pathways.

### Quantitative real-time PCR

The reaction mixture contained 10 μl KAPA SYBR FAST qPCR Master Mix (KAPA Biosystems, # K4601), 2 μl 20-fold diluted cDNA, 0.4 μM of each forward and reverse primer (**Supplementary Table S1**), and ddH2O in a final volume of 20 μl. Amplifications were performed with the following program: 95°C for 3 s and 40 cycles of 95°C for 10 s, 60°C for 30 s, and 72°C for 3 s. No-template reactions were used as negative controls, and *PP2A-2* was the reference gene. The quantitative real-time PCR was performed on the LightCycler^®^ 480 System (Roche Molecular Systems, Germany). Each sample was assessed in four technical replicates, and the data were analyzed using −2^−△△Ct^ method.

## Results

### Leaf phenotypes of *Ginkgo biloba* “SongZhen”

“SongZhen” is *G. biloba* with variant leaf types including needle-shaped, trumpet-shaped, strip-shaped, deeply split semi fan-shaped, and normal fan-shaped leaves, and these leaf types appear at the same time on clonal individuals of “SongZhen” (**Supplementary Figure S1A**). The fan-shaped leaves similar to ordinary *Ginkgo* leaves are defined as “normal leaf, NL” and the leaves with other types as “abnormal leaf, ANL.” The normal leaves and abnormal leaves were mostly clustered on different short branches (**Supplementary Figures S1B, C**). Therefore, the apical buds of the short branches with normal leaves or abnormal leaves were separately collected for the transcriptome analysis.

### Morphology and tissue structure of “SongZhen” leaves

**Figure 1:**
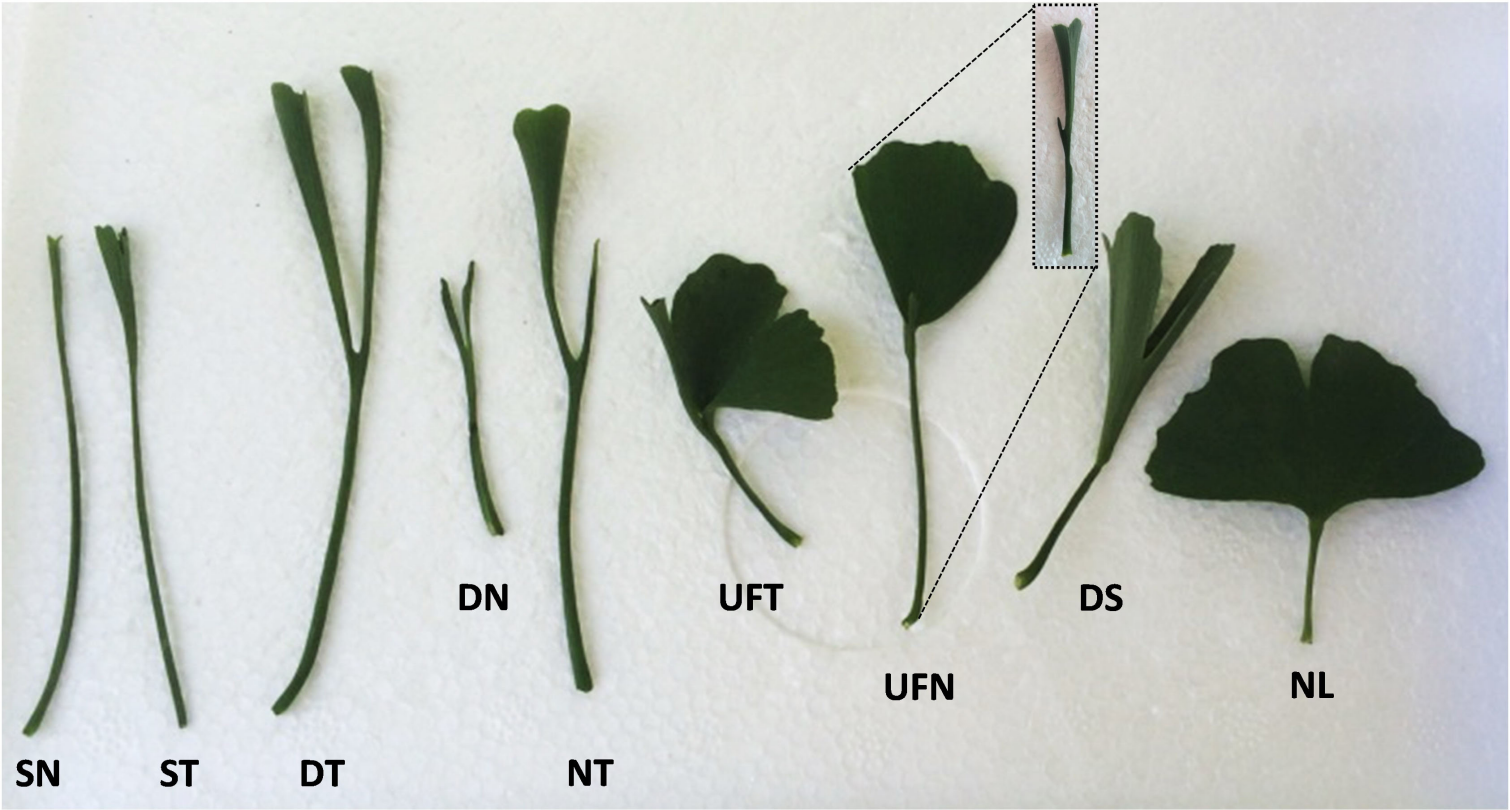
The different types of leaves of *Ginkgo biloba* “SongZhen.” SN, single-needle; ST, single-trumpet; DT, double-trumpet; DN, double-needle; NT, needle and trumpet; UFT, unfolded and trumpet; UFN, unfolded and needle; DS, deep-split; NL, normal leaf. The small figure in the dotted box is a side view of UFN leaf.

According to the variation degree, the leaves of “SongZhen” were mainly divided into nine types (**Figure 1**): single-needle, single-trumpet, double-trumpet, double-needle, needle and trumpet, unfolded and trumpet, unfolded and needle, deep-split, and normal leaf. The normal leaf was a typical modern ginkgo leaf in a fully developed fan shape. Except for the single-needle and single-trumpet leaves, the other variant leaves were basically separated into two parts from the petiole, one part was folded into a needle or trumpet shape, and the other part was expanded into fan shape, or both two parts formed needles or trumpets shape. The deep-split leaf was a partial fusion of two unfolded leaves at the bottom of the blade (**Figure 1**).

**Figure 2:**
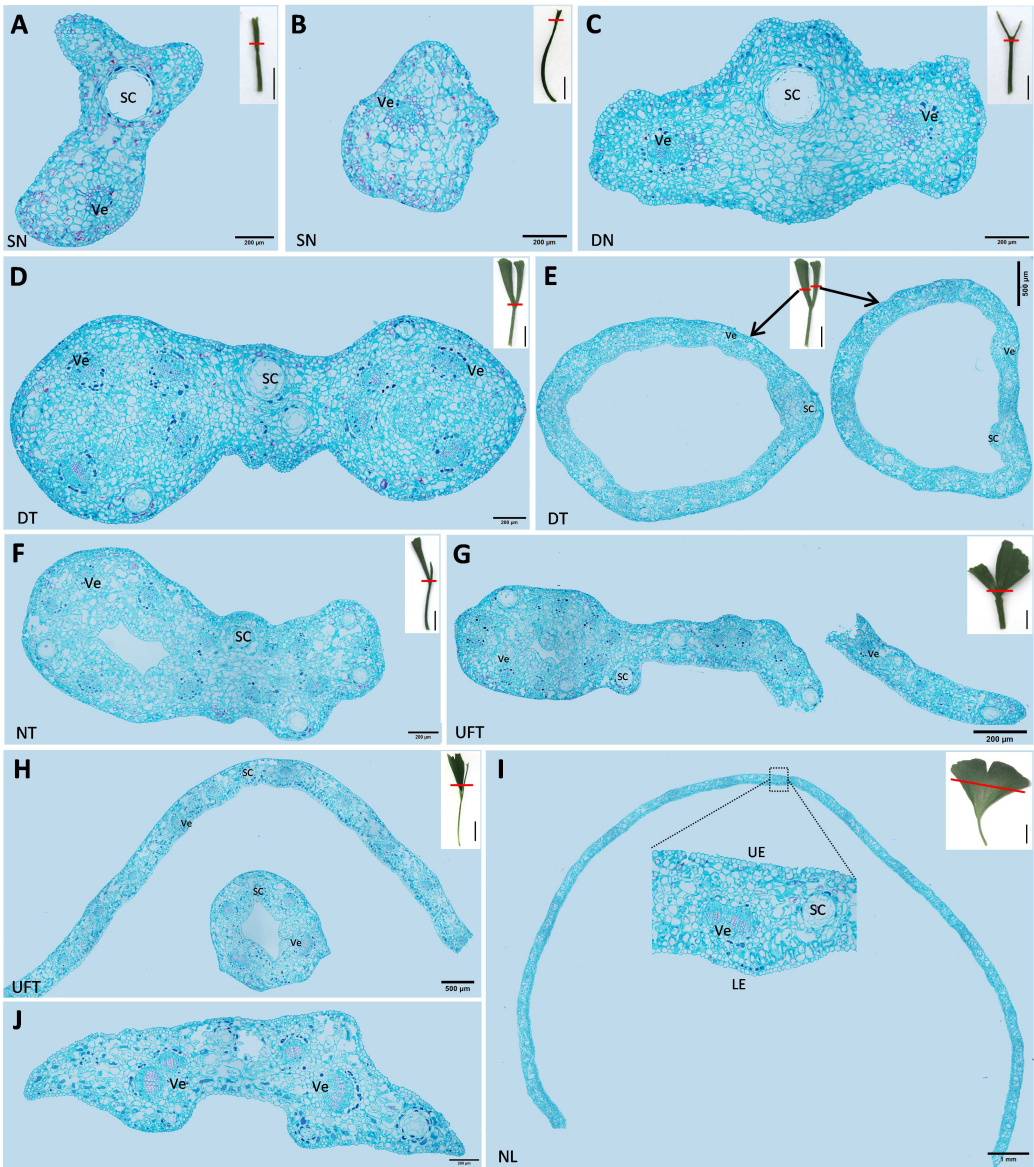
The cross-sections of different types of leaves of *Ginkgo biloba* “SongZhen.” **(A, B)** Single-needle (SN) leaf; bar, 200 µm. **(C)** Double-needle (DN) leaf; bar, 200 µm. **(D)** The base part of double-trumpet (DT) leaf; bar, 200 µm. **(E)** The upper part of double-trumpet (DT) leaf; bar, 500 µm. **(F)** Needle and trumpet (NT) leaf; bar, 200 µm. **(G)** Unfolded and trumpet (UFT) leaf (unfold and trumpet part fused together; bar, 500 µm). **(H)** Unfolded and trumpet (UFT) leaf (unfold and trumpet part separated; bar, 500 µm). **(I)** Normal leaf (NL); bar, 1 mm. **(J)** The petiole of normal leaf; bar, 200 µm. Sc, secretory cavity; Ve, vein; UE, upper epidermis; LE, lower epidermis. The small pictures at the upper right corner of panels **(A–I)** are the complete leaves, where the red line shows the slicing position, and the black line is the scale; bar, 1 cm.

The cross-sections of different types of leaves in “SongZhen” were prepared. The single-needle leaves had secretory cavity (SC) and vein (**Figure 2A**), or vein only (**Figure 2B**). In double-needle leaves, there were one SC and two veins at the fusion part of the bottom of the leaf, and the SC was located at the ridge, while two veins were on both sides of the SC (**Figure 2C**). In double-trumpet leaves, two rings were formed at the junction of the leaf and petiole where the two trumpets fused (**Figure 2D**). Then, the two rings could develop and split into two larger rings (**Figure 2E**). Needle type was the extreme for the undeveloped leaf, and other leaves were between it and the normal leaf type. For example, the needle and trumpet leaves consisted one needle- and one trumpet-type leaf (**Figure 2F**), while the unfolded and trumpet leaves had one side with half unfolded normal leaf and another side with trumpet-type leaf (**Figures 2G, H**). In contrast, the normal leaves were the same as wild-type *G. biloba* (Wang et al., 2011), which were long and flat, and composed of epidermis, mesophyll, vein, and secretory cavity (**Figure 2I**).

**Figure 3:**
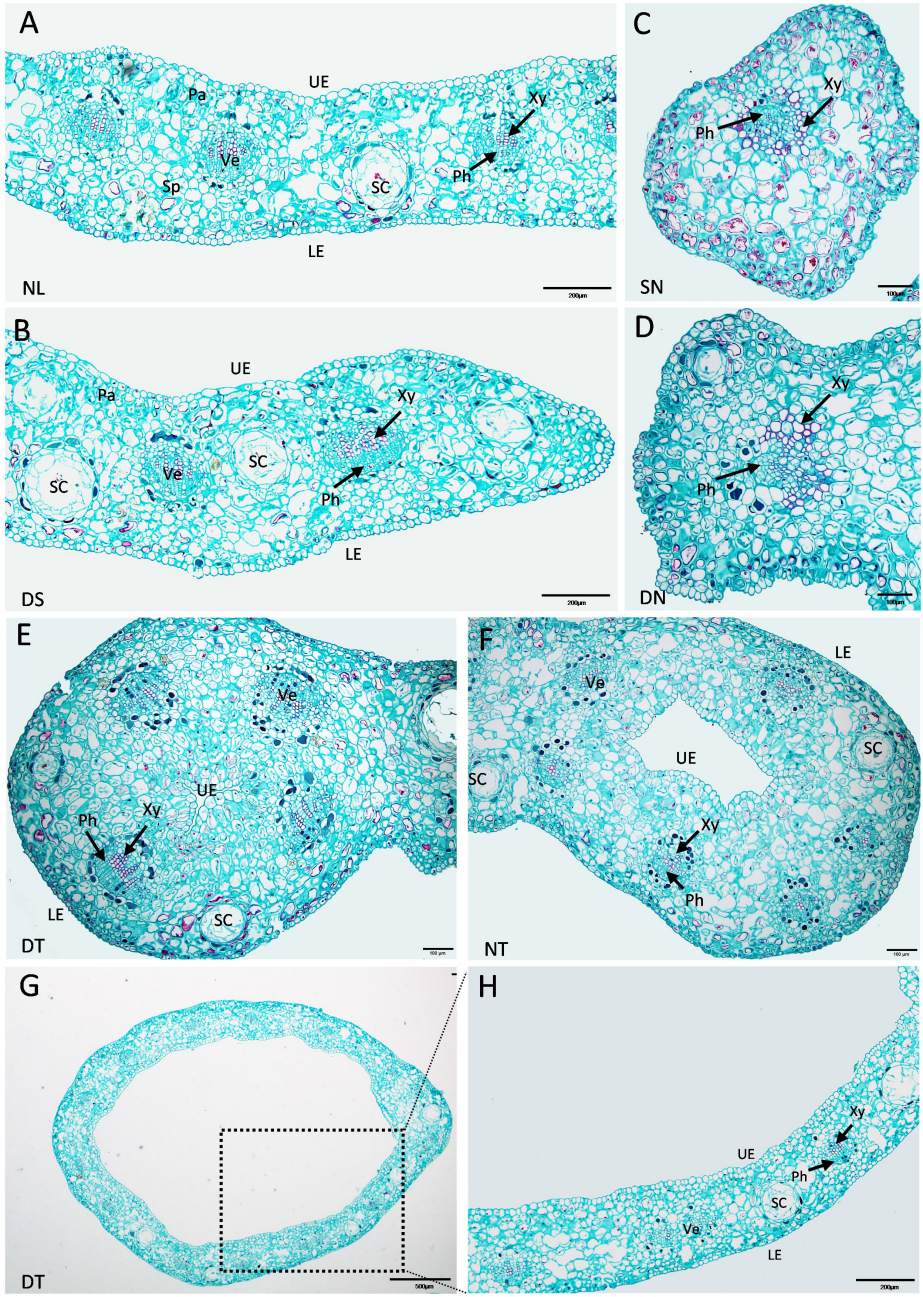
Enlarged cross-sections of leaves of *Ginkgo biloba* “SongZhen.” **(A)** Normal leaf (NL); bar, 200 µm. **(B)** The leaf margin of the deep-split (DS) leaf; bar, 200 µm. **(C)** Single-needle (SN) leaf; bar, 100 µm. **(D)** One side of double-needle (DN) leaf; bar, 100 µm. **(E)** One side of double-trumpet (DT) leaf; bar, 100 µm. **(F)** The trumpet side of needle and trumpet (NT) leaf; bar, 100 µm. **(G)** The trumpet leaf; bar, 500 µm. **(H)** The enlarged view of the black dotted box in panel **(G)**; bar, 200 µm. Sc, secretory cavity; Ve, vein; UE, upper epidermis (adaxial side); LE, lower epidermis (abaxial side); Xy, xylem; Ph, phloem; Pa, palisade tissue; Sp, spongy tissue.

The enlarged images from the cross-section of “SongZhen” leaves in **Figure 2** show that the normal leaves of “SongZhen” had a similar tissue structure with that of wild-type *G. biloba* (Wang et al., 2011). The upper and lower epidermis had only one layer of cells. The palisade tissue was composed of one to two layers of cells and arranged closely, while the sponge tissue was irregular and arranged loosely. In the mesophyll, veins were evenly distributed and alternated with the secretory cavity. The leaf vein was a complete vascular bundle with xylem on the adaxial side and phloem on the abaxial side (**Figures 3A, B**). On the other hand, single- and double-needle leaves were not typical leaves without obvious palisade and spongy tissues. The veins were not normal vascular bundle, where the phloem cells were scattered and surrounded by xylem cells (**Figures 3C, D**). The shape of single- and double-trumpet leaves were not flat and curled inward to form a loop from the abaxial side to adaxial side, i.e., the upper epidermis was inside, and the lower epidermis was outside (**Figures 3E, F**). The veins were normal vascular bundles, with xylem on the adaxial side and phloem on the abaxial side (**Figures 3E–H**). From the above description, the abnormal leaves could be all due to their incomplete unfolding, whose structure is more likely due to abaxialization.

### Transcriptome change in ANL buds

To investigate the genetic basis for the ANL development, we performed transcriptome analysis. Six transcript libraries from the NL buds (NL1, NL2, and NL3) and ANL buds (ANL1, ANL2, and ANL3) were constructed and analyzed by high throughout RNA-seq. With the process of quality control for raw data, 65,905,424, 87,418,414, 98,233,334, 136,008,800, 72,083,256, and 65,922,944 clean reads were obtained from these libraries with Q30 above 85.20%. The clean data were mapped to the *G. biloba* genome (Guan et al., 2016), and the alignment ratio of samples ranges from 93.41% to 94.26% (**Supplementary Table S2**). Based on the comparison, 1,796 novel genes were discovered, of which 1,038 genes were annotated.

The transcript abundance of each gene from NL and ANL data were normalized into FPKM. Compared with NL, a total of 3,762 significant difference expression genes (DEGs) were identified in ANL libraries with the threshold of fold change ≥2 and FDR ≤ 0.05 (**Supplementary Table S3**), including 1,391 upregulated and 2,371 downregulated genes (**Supplementary Figure S2**). Among these identified DEGs, 1,917 DEGs were annotated with 21 biological processes, 16 cellular component, and 13 molecular functions in GO categories, and significantly enriched (p ≤ 0.01) into 76 GO terms (**Supplementary Table S4**). Among downregulated genes, the term of “cellular glucan metabolic process,” “oxidation–reduction process,” “response to desiccation,” “response to stress,” and “lignin catabolic process” were the dominant groups in the biology process (**Supplementary Figure S3A**), while the upregulated genes mainly focused on “nucleosome assembly,” “cell proliferation,” and “flavonol biosynthetic process” (**Supplementary Figure S3B**). Furthermore, 3,762 significantly DEGs were compared with KEGG database to analyze their biology pathway. The 276 downregulated genes were enriched to 84 KEGG pathway (**Supplementary Table S5**), of which the most significant ones were “phenylpropanoid biosynthesis,” “cutin, suberine, and wax biosynthesis,” “flavonoid biosynthesis,” and “plant hormone signal transduction” (**Supplementary Figure S4A**). While 76 enriched pathways were identified from 161 upregulated genes (**Supplementary Table S5**), and “betalain biosynthesis,” “arginine biosynthesis,” and “nitrogen metabolism” were the top 3 (**Supplementary Figure S4B**). The significant change in GO terms and KEGG pathway indicates the significant reprogramming of gene expression in ANL development.

### The genes related to leaf formation

**Figure 4:**
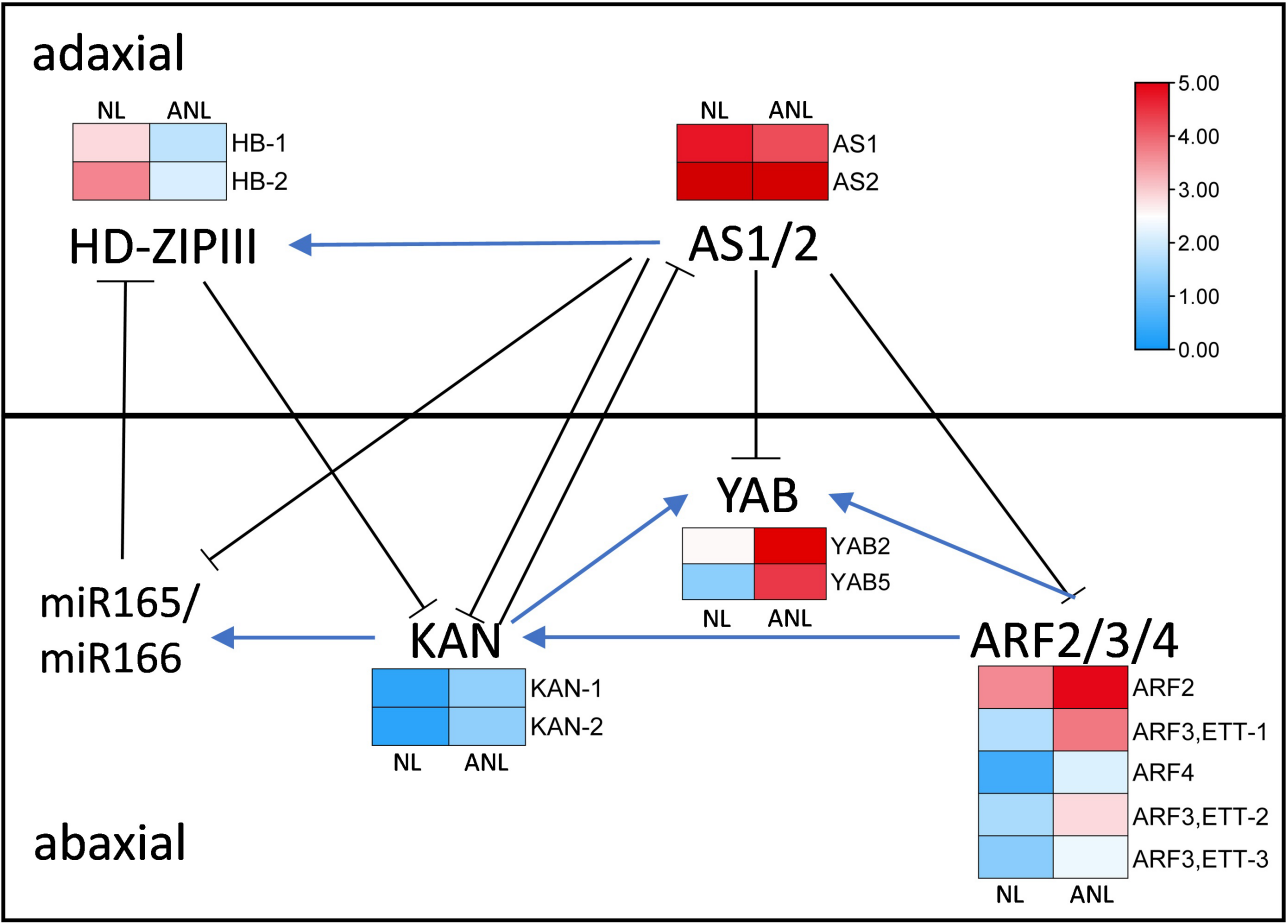
The gene regulatory network on leaf adaxial–abaxial development with their expression change in NL and ANL buds. AS, asymmetric leaf; HD-ZIPIII, homeodomain-leucine zipper III; ARF, AUXIN RESPONSE FACTOR; YAB, YABBY; KAN, KANADI; miR165/166, microRNA165/166.

The anatomical structure of the trumpet-type leaves was similar to abaxialized leaves in the dicotyledons, and needle-type leaves might have resulted from severe abaxialization of leaf development. The adaxial–abaxial prepattern of leaf might be established prior to leaf initiation, and the adaxial–abaxial polarity genes had prepattern expressed at the PZ prior to leaf primordium formation (Yu et al., 2017). Therefore, we screened the adaxial–abaxial polarity genes in *Arabidopsis* that are mainly involved in the formation and maintenance of adaxial–abaxial polarity of leaves to find out their homologous genes in *G. biloba*. The differentially expressed genes (**Supplementary Table S6**) were assigned with their expression values in the regulatory network on the leaf adaxial–abaxial development (**Figure 4**).

Three *ARFs* (*ETTIN* (*ARF3*), *ARF4*, and *ARF2*), *KAN*, and *YAB* genes are expressed in the abaxial domain and redundantly promote abaxial identity. There were five *ARF*, two *KAN*, and two *YAB Ginkgo* homologous genes that were higher expressed in ANL buds, indicating that the increasing expression values of these abaxial dominant genes would promote leaf abaxial development. The *HD-ZIPIII* and *ASYMMETRIC LEAF1* genes are predominantly expressed on the adaxial domain and can promote adaxial cell fate. Two *Ginkgo HB* homologous genes exhibited lower expression values in ANL than in NL buds, while the expression of *AS1* and *AS2* homologous genes slightly decreased in ANL buds. The adaxially localized *AS1–AS2* complex negatively regulates the expression of *ETT*, *ARF*4, *KAN*, and *YAB* (Iwasaki et al., 2013) but positively regulates *HD-ZIPIII* expression (Fu et al., 2007). Therefore, the decreased expression of these adaxial dominant genes might weaken their promotion on leaf adaxial domain while reducing their inhibition on abaxial dominant genes, leading to the enhanced development of abaxial domain in ANL.

**Figure 5:**
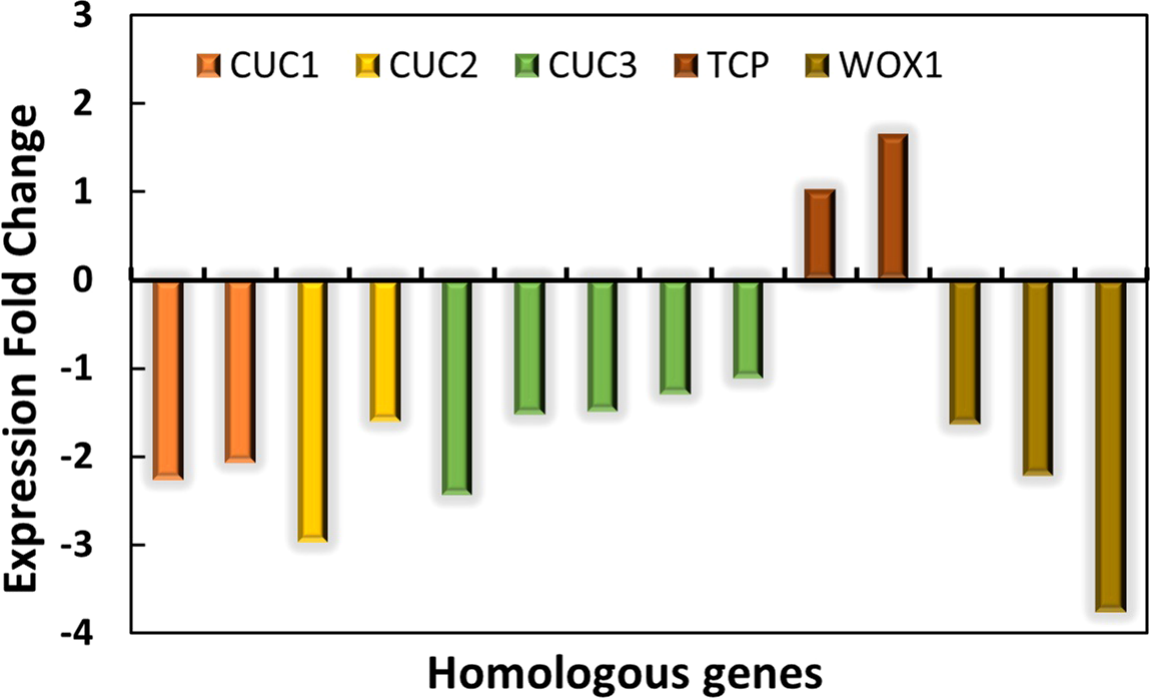
The expression changes in *CUC*, *TCP*, and *WOX* genes in ANL buds compared with NL buds.

The normal leaves of “SongZhen” were fully expanded fan-shaped blades, while most of the abnormal leaves were smaller and narrower than normal ones. The abnormal leaves were divided into two parts from the leaf stalk, in which the expanded side was half fan shaped, and the unfolded side was fused into trumpet or into more serious needle shaped. The *CUC* and *TCP* transcription factors are crucial for boundary formation and margin separation. The homologous *CUC1*, *CUC2*, and *CUC3* genes exhibited lower expression, while the two *TCP* genes showed higher expression in ANL buds (**Figure 5**). This indicated that the formation of needle-type, trumpet-type, and deep-split leaves in ANL might be due to the downregulation of *CUC* genes and upregulation of *TCP* genes, which limited the tissue development and separation. The *WOX* genes are expressed at the adaxial–abaxial boundary layer, where they can promote leaf blade outgrowth. In ANL buds, the expression values of *WOX1* genes were lower compared with that in NL buds. This might also explain why the abnormal leaves are smaller and narrower compared to the normal leaves.

### The expression of DEGs in abnormal leaves

**Figure 6:**
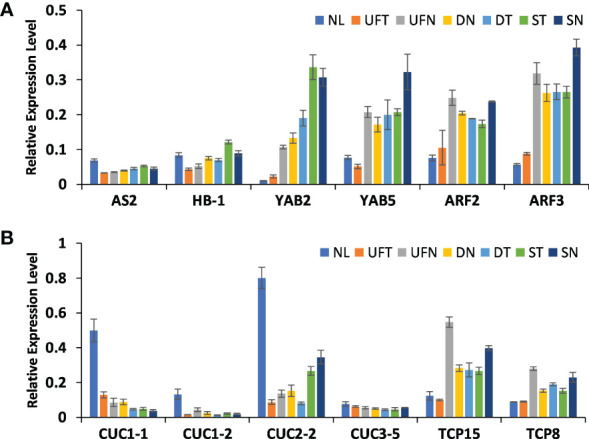
The relative expression profiles of genes in abnormal and normal leaves. The relative expression levels of genes related to **(A)** leaf adaxial–abaxial formation and **(B)** leaf boundary formation. NL, normal leaf; UFT, unfolded and trumpet leaf; UFN, unfolded and needle leaf; DN, double-needle leaf; DT, double-trumpet leaf; ST, single-trumpet leaf; SN, single-needle leaf.

In order to determine whether the expression levels of above genes in different morphological leaves of “SongZhen” were related to the variation degree of abnormal leaves, quantitative PCR was performed in the collected samples of six different leaf types, including normal leaf, unfolded and trumpet leaf, unfolded and needle leaf, double-trumpet leaf, double-needle leaf, single-trumpet leaf, and single-needle leaf according to the degree of leaf variation. The gene expression in abnormal and normal leaves was consistent with that in buds (**Figure 6**). The abaxial dominant genes, such as *YAB2*, *YAB5*, *ARF2*, and *ARF3*, were highly expressed in abnormal leaves, and the expression levels increased with the degree of leaf variation, especially *YAB2* whose expression values were increased gradually from NL to SN. In contrary, the expression levels of *AS2* in different types of abnormal leaves were similar, but they all were lower than that in normal leaves, and *HB-1* was lowly expressed from UNT to DT accordingly (**Figure 6A**). In abnormal leaves, the expression levels of *CUC* genes were much lower than that of normal leaves, and their expression values reduced gradually from UNT to SN except *CUC2-2*, while the *TCP* genes were highly expressed in needle and trumpet leaves (**Figure 6B**). This indicates that these genes related to adaxial–abaxial formation and boundary development might affect the formation of abnormal leaves in “SongZhen.”

## Discussion

The development of the leaf is mainly through the formation and maintenance of polarity of the medial–lateral, proximal–distal, and adaxial–abaxial axis to achieve the three-dimensional structure, which is determined by a complex network of genes. *Ginkgo biloba* has a typical dichotomous bifurcate leaf structure, while different leaf types of the *G. biloba* variety “SongZhen” provide materials to access the mechanism of the formation of variant leaf types of *G. biloba*, which may shed light on the unique leaf development of *G. biloba* in an evolutionary view.

The variant materials of “SongZhen” could reflect the leaf evolution in *Ginkgo*. The double-needle- and double-trumpet-shaped leaves are similar to that of the Middle Jurassic *Ginkgo* leaf fossils unearthed in Uzbekistan (Nosova, 2013) from coniferous *Ginkgo* dated back about 200 million years ago. According to the telome theory, the leaves of *G. biloba* had undergone a series of evolution from dichophyllum with two main veins, to *Trichopitys* having the hairy leaves with four main veins, then the hairy leaves became thin and lobed type *Baiera*, and finally evolved into the four-lobe type *Ginkgoites* sp., which was the most recent ancestor to the modern *G*. *biloba* leaves (Zhou and Zheng, 2003; Zhou, 2006). There are paired vascular bundles transiting from the stem to the petiole, and each vascular bundle in the petiole gradually produces bifurcated branches to form four parallel vascular bundles; then, the four vascular bundles in the petiole produce three- and four-level bifurcated branches in *Ginkgo* leaves (Zhou, 2006). The double-needle (trumpets) leaf, needle and trumpet leaf, unfolded and needle (trumpet) leaf, or deep-split leaf in “SongZhen” have the vascular bundles joined together in the petiole but divided into two parts in the blade, which can be seen from the cross-section of the base of the double-trumpet leaves (**Figure 2D**) and of trumpet leaf with the separated vascular bundles (**Figure 2E**). The variant leaf types of “SongZhen” have the characteristics of *dichophyllum*, *Trichopitys*, and *Baiera*, having bifurcated, deeply split, or unfolded leaves. Therefore, it is speculated that the appearance of “SongZhen” may be a kind of atavism caused by bud mutation. The needle-like and the fused vascular bundles in abnormal leaves are regarded as “atavism” (Bauer et al., 2013; Nosova, 2013). The failure of adaxial–abaxial polarity formation and boundary separation are probably the reason to give rise to needle (trumpet) leaves with abaxialization and boundary fusion.

The most obvious variation of “SongZhen” were needle- and trumpet-type leaves. It can be seen from the transverse anatomy that the trumpet-type leaves form one or two closed rings on both sides of the central axis of the leaves, but they have a complete leaf structure with the upper epidermis in the inner ring and the lower epidermis in the outer ring. This is similar to the abaxialized leaves in dicotyledons, with promoted development of the abaxial domain to surrounding the adaxial domain. The needle-type leaves are the most severe case, which are similar to *phan* mutant in *Antirrhinum* and *as1/as2* double mutant in *Arabidasis* that lack laminae and adaxial cell types (Xu et al., 2003). It can be inferred that the development of the abaxial region of abnormal-type leaves may be greater than the adaxial region or even no adaxial region.

At present, the research on leaf polarity development is mainly focused in *Arabidopsis*, but the expression and function of the adaxial- and abaxial-domain-promoting genes may be diversified among various species. There are four *KANADI* paralogs in *Arabidopsis* with *KAN1* and *KAN2* specifying in abaxial leaf identity. Loss-of-function *kan1* and *kan2* mutants develop narrow cotyledons and leaves with ectopic outgrowths on their abaxial side and display adaxialized lateral organs, while *KAN1* and *KAN2* overexpression causes abaxialization and SAM termination (Eshed et al., 2001; Kerstetter et al., 2001). Phylogenetic analysis and *in situ* hybridization showed that the KAN function in leaf polarity is likely conserved across ferns, gymnosperms, and angiosperms except lycophyte (Zumajo-Cardona et al., 2019). In addition, severe polarity defects were observed in *Arabidopsis ett-1 arf4-1* and *ett-1 arf4-2* double-mutant plants resulting in abaxialized leaves similar to *kan1 kan2* mutants (Pekker et al., 2005). In fact, ARF and KAN proteins may form complexes and play a synergism role in promoting abaxial region differentiation (Kelley et al., 2012). The YABBY transcription factor family is expressed on the abaxial side of lateral organs and redundantly promote abaxial identity in *Arabidopsis*. Loss of function of two redundant *YAB* genes, namely, *FILAMENTOUS FLOWER* (*FIL*) and *YABBY3* (*YAB3*), results in narrow leaves and a partial loss of abaxial cell identity, whereas ectopic expression of *FIL* or *YAB3* in leaves and petals causes partial abaxialization (Siegfried et al., 1999; Eshed et al., 2001). However, the *YAB* gene expression is adaxialized in maize (Juarez et al., 2004) and is not polarized in rice (Yamaguchi et al., 2004), wheat (Zhao et al., 2006), soybean (Yang et al., 2019), and bamboo (Liu et al., 2020). Ectopic expressions of wheat *TaYAB1* and soybean *GmFILa* in *Arabidopsis* both cause the partial abaxialization of the adaxial epidermises of leaves (Zhao et al., 2006; Yang et al., 2019). These results indicate that the expression patterns and function of *YAB* genes have diverged between monocots and dicots. In ANL buds, *KAN* (*KAN1* and *KAN2*), *ARF* (*ETT*, *ARF2*, and *ARF4*), and *YAB* genes were upregulated. The higher expression of these abaxial domain-promoting genes in ANL buds and abnormal leaves might promote the overdevelopment of the abaxial domain of abnormal leaves, and the adaxial mesophyll cells are replaced by abaxial cells that leads to abaxialization leaves of “SongZhen.”

*HD-ZIPIII* expression is sufficient to define adaxial cell fate, and they have conserved functional roles in *Arabidopsis*, rice, maize, and most likely across angiosperms (Dkhar and Pareek, 2014). Dominant gain-of-function *phb*, *phv*, and *rev* mutants show loss of abaxial identity and adaxialization of leaves, while loss-of-function *phb phv rev* triple-mutant plants form severe abaxialized leaves and exhibit loss-of-SAM phenotypes in *Arabidopsis* (McConnell et al., 2001; Emery et al., 2003). Two *HB* genes had lower expression values in ANL buds, and *HB-1* was also lowly expressed in abnormal leaves, suggesting that the abnormal leaf development was abaxialized. Adaxial cell fate is also promoted by *AS2.* The overexpression of *AS2* in *Arabidopsis* resulted in plants with narrower curly leaves displaying dramatic alteration in the identity of both adaxial and abaxial epidermal cells, and the abaxial side showed mostly adaxial features (Xu et al., 2003). The expression values of *AS2* had lower expression in abnormal leaves than normal ones, which further caused the abaxialization of “SongZhen” leaves. Therefore, *HD-ZIPIII* and *AS1*–*AS2* that had lower expression in ANL buds and abnormal leaves will weaken their inhibition on genes specified in the leaf abaxial side and eventually lead to the leaf abaxialization. To sum up, the expression of adaxial–abaxial polarity genes in “SongZhen” was similar to that in *Arabidopsis*, indicating that these genes may play a decisive role in leaf development, thus showing some conservatism between gymnosperms and angiosperms. However, the regulatory mechanism in leaf development among different species remains to be further studied.

Boundary separation is probably hurdled in single- or double-needle (trumpet) leaves in “SongZhen.” The low expression of *CUC*s and high expression of *TCPs* in ANL may explain this type of development. *cuc1 cuc2* double mutation causes a complete lack of embryonic shoot meristem formation and a fusion of the cotyledons (Aida et al., 1999; Hibara et al., 2006). *CUC3* also participates in embryonic shoot meristem formation and cotyledon boundary formation redundantly with *CUC1* and *CUC2* (Vroemen et al., 2003). Furthermore, inactivation of TCP function in *Arabidopsis* (Efroni et al., 2008), or its orthologs CINCINNATA (CIN) in *Antirrhinum* (Nath et al., 2003) and LANCEOLATE (LA) in tomato (Ori et al., 2007), causes overproliferation at the leaf margin, which leads to expanded, crinkly leaves or leaflets with serrated margin. In addition, overexpression of TCP3 suppresses the expression of *CUC* genes and results in the fusion of cotyledons and defects in formation of shoots (Koyama et al., 2007; Koyama et al., 2017). In ANL buds, the low expression of *CUC*s while the high expression of *TCP*s would affect the boundary formation in “SongZhen” with needle or fused trumpet-type leaves. Quantitative PCR results also showed that the expression of *CUC* genes in abnormal leaves was much lower than that in normal ones, while the expression of *TCP* genes was higher in abnormal leaves, indicating that *CUC* and *TCP* play an important role in the formation of abnormal leaves of “SongZhen.”

Overall, the leaf phenotype of “SongZhen” variety is heritable. The plants obtained through grafting can have these abnormal leaf types continuously for more than years. However, the appearance and amount of these abnormal leaves are random, and the leaf type on a long branch may change in different years, although the abnormal and normal leaves are always clustered on different short branches, respectively. This indicates that the environment fluctuation may influence gene expression, leading to the phenotypic change. Transcriptome data in this study also show that “oxidation reduction process,” “response to desiccation,” and “response to stress” are the main biological processes among downregulated genes, indicating that “SongZhen” mutants are more vulnerable to the physiological and environmental changes, resulting in the randomness and variability of genetic variation. The results of microscopy and transcriptome analysis showed that the abnormal leaves of *G*. *biloba* “SongZhen” are mainly caused by the imbalance of abaxial and adaxial development of leaf and the impairment of leaf boundary. The genes related to abaxial domain are highly expressed, while the expression levels of adaxial-domain promoting genes are decreased in ANL buds. In addition, the low expression of genes related to leaf boundary development in ANL buds indicates that single- or double-needle (trumpet) leaves may be due to the leaf tissue fusion. This study provides an insight into the mechanism of the development of the abnormal leaves in “SongZhen” and the hint in evolution of *G*. *biloba* leaves.

## Data availability statement

The original contributions presented in the study are publicly available. This data can be found here: NCBI, PRJNA896381.

## Author contributions

ML and YW designed the study. FT and PS collected the tissues used for this study and analyzed the data. FT completed the section and microscopic observation of different types of leaves in “SongZhen.” FT drafted the manuscript, and ML revised it. QZ and FZ gave valuable suggestions to the manuscript. All authors contributed to the article and approved the submitted version.
