# Supplementary material for: Insight into the formation of trumpet and needle-type leaf in Ginkgo biloba L. mutant
Source: Front Plant Sci. 2022 Dec 9;13:1081280. doi: 10.3389/fpls.2022.1081280 (PMC9780455; doi:10.3389/fpls.2022.1081280)
Supplement: Supplementary file 1 [file DataSheet_1.pdf]

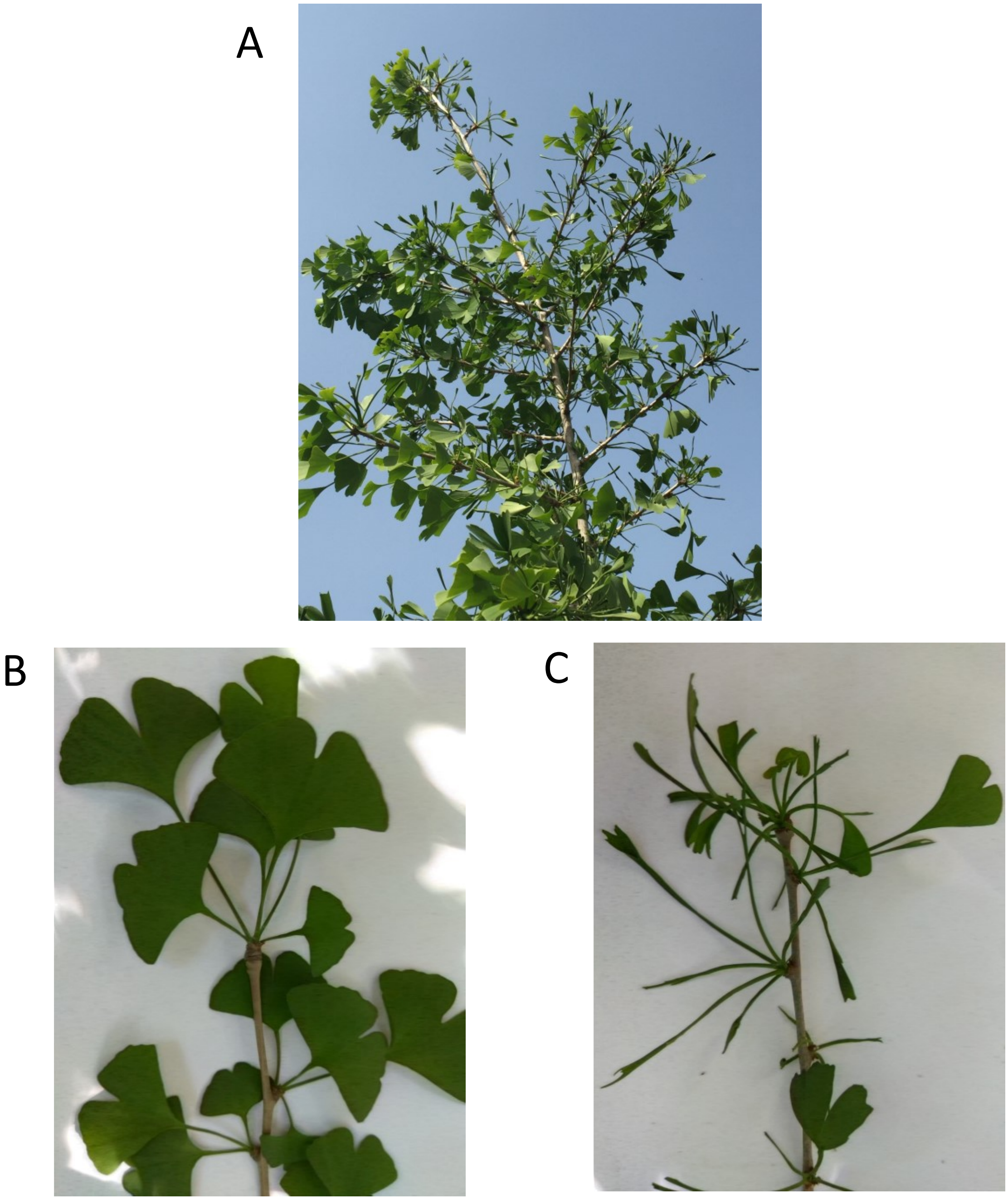

Figure S1. The branches of *Ginkgo biloba* 'Songzhen'

(A) The normal and abnormal leaves on the long branches; (B) The normal leaves on the short branches; (C) The abnormal leaves on the short branches.

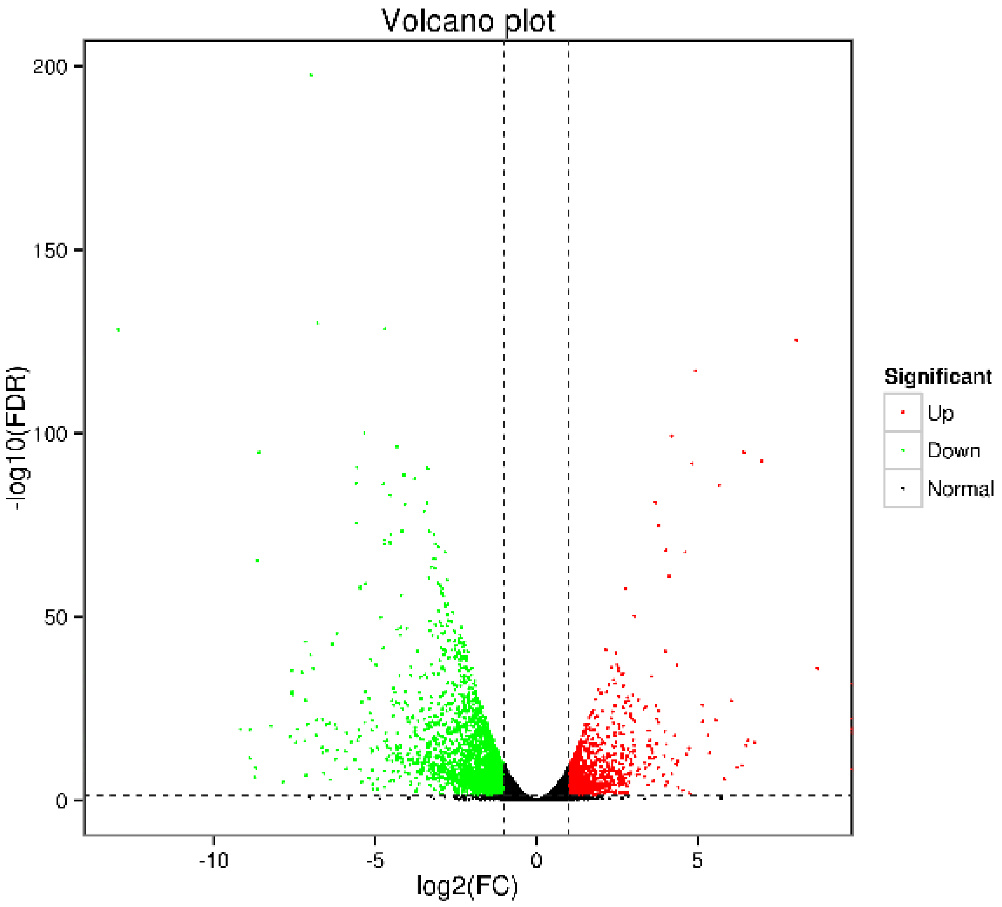

Figure S2. The expression profiles of the identified DEGs.

Each dot represented a gene. Abscissa represented the fold change of the FPKM change of NL vs. ANL libraries; ordinate represented the negative logarithm of p-value. The red and green dots represented the significant EDGs with Fold FDR≤0.05 and $\log_2$(fold change)≥1, and black ones showed those non-differentially expressed genes.

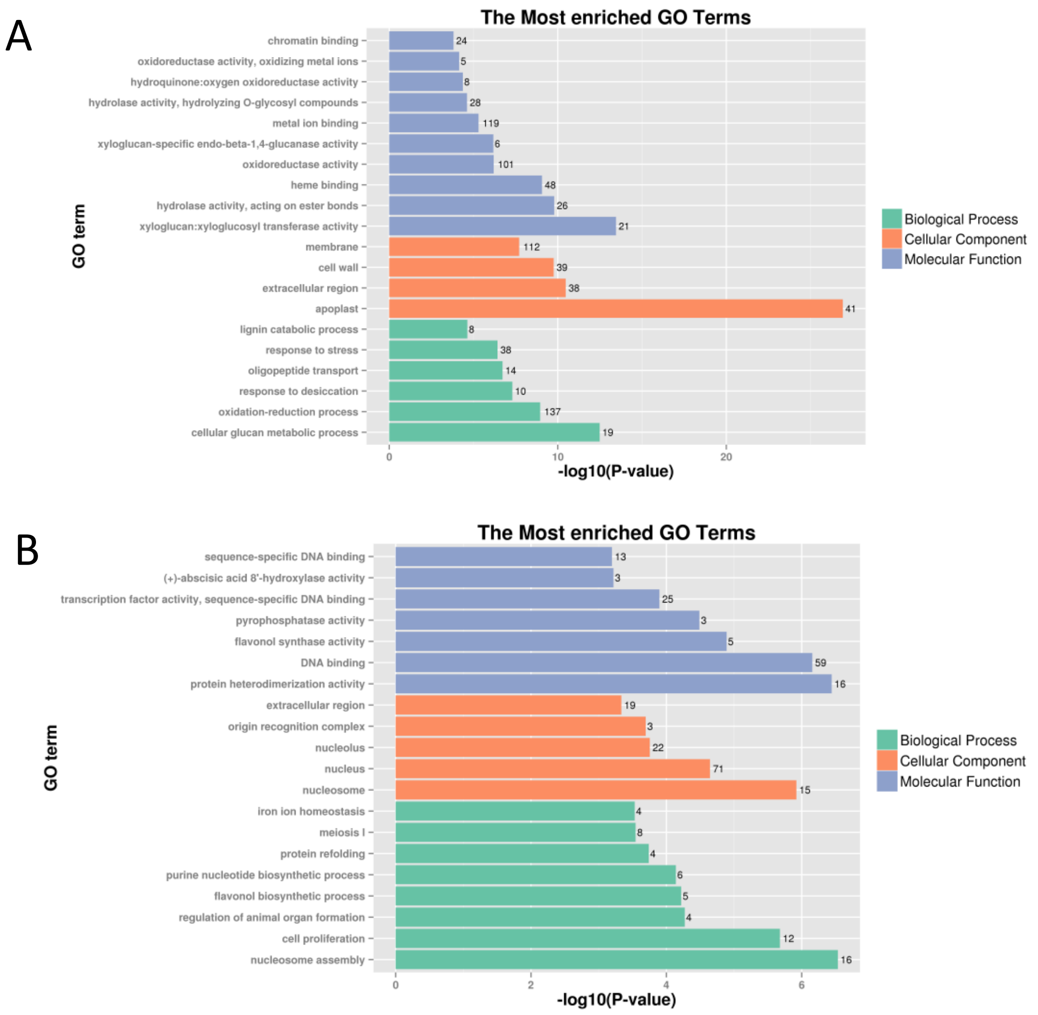

Figure S3. The most enriched GO terms of differentially expressed genes.

The most enriched GO terms of (A) down-regulated genes; (B) up-regulated genes. Go terms contains three main branches of biological processes, molecular function and cellular components.

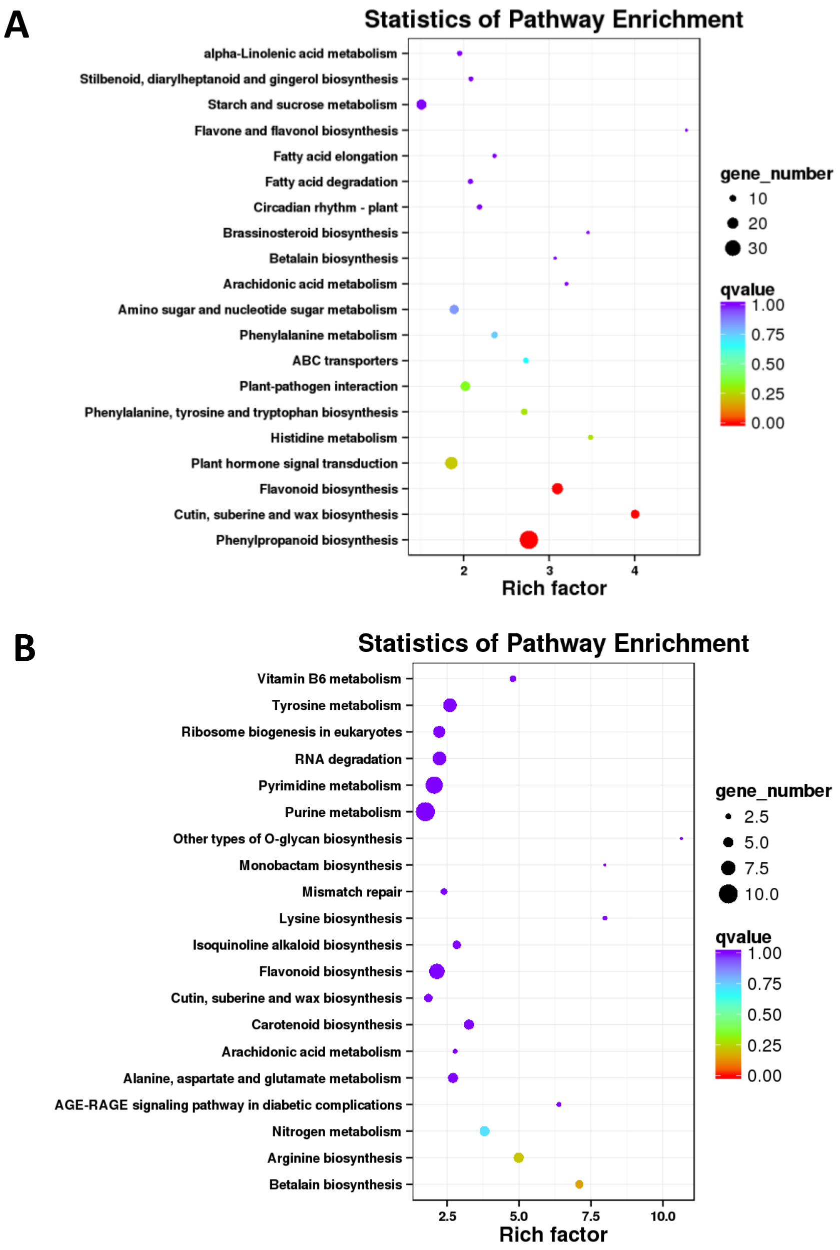

Figure S4. KEGG pathway enrichment bubble diagram of differentially expressed genes

The most enriched KEGG pathway of (A) down-regulated genes; (B) up-regulated genes. In the figure, each circle represents a KEGG pathway. The ordinate is the name of the pathway, and the abscissa is the enrichment factor, which represents the ratio of the gene proportion annotated to a pathway in the differential gene to that annotated to the pathway in all genes. The larger the enrichment factor, the more significant the enrichment level of the differentially expressed genes in this pathway.
